# Using practice facilitation to improve alcohol-related care in primary care: a mixed-methods pilot study protocol

**DOI:** 10.1186/s13722-022-00300-x

**Published:** 2022-03-14

**Authors:** Rachel L. Bachrach, Matthew Chinman, Keri L. Rodriguez, Maria K. Mor, Kevin L. Kraemer, Cécile E. Garfunkel, Emily C. Williams

**Affiliations:** 1 Center for Health Equity Research and Promotion, VA Pittsburgh Healthcare System, Research Office Building (151R-U), University Drive C, Pittsburgh, PA 15240-1001 USA; 2 Mental Illness Research, Education, and Clinical Center, VA Pittsburgh Healthcare System, Research Office Building (151R-U), University Drive C, Pittsburgh, PA 15240 USA; 3 Department of Psychology, University of Pittsburgh, Pittsburgh, PA 15260 USA; 4 The RAND Corporation, 4570 Fifth Avenue, Suite 600, Pittsburgh, PA 15213 USA; 5 Deparatment of Biostistic, Graduate School of Public Health, University of Pittsburgh, Pittsburgh, PA 15261 USA; 6 Division of General Internal Medicine, Department of Medicine, University of Pittsburgh, Pittsburgh, PA 15261 USA; 7 Division of Geriatrics, Department of Medicine, University of Pittsburgh, Pittsburgh, PA 15261 USA; 8 Department of Health Systems and Population Health, University of Washington School of Public Health, Seattle, WA 98195 USA; 9 Health Services Research & Development (HSR&D) Center of Innovation for Veteran-Centered and Value-Driven Care, Veterans Affairs (VA) Puget Sound Health Care System, Seattle, WA 98108 USA

**Keywords:** Alcohol, Screening, Brief intervention, Alcohol use disorder treatment, Practice facilitation, Implementation intervention

## Abstract

**Background:**

Alcohol use is a significant risk factor for disability and death in U.S. adults, and approximately one out of every six Veterans seen in primary care (PC) report unhealthy alcohol use. Unhealthy alcohol use is associated with increased risk for poor medical outcomes, substantial societal costs, and death, including suicide. Based on substantial evidence from randomized controlled trials and the U.S. Preventive Services Task Force, VA/DoD clinical guidelines stipulate that all Veterans screening positive for unhealthy alcohol use should receive evidence-based alcohol care in PC, including brief counseling interventions (BI) and additional treatment (e.g., pharmacotherapy) for those with alcohol use disorders (AUD). The VA pioneered implementing alcohol screening and BI in PC, yet substantial implementation gaps remain. To improve alcohol-related care, this study will conduct a pilot study to assess whether a multi-faceted evidence-based implementation strategy—practice facilitation—has the potential to improve PC-based alcohol-related care at a single VA clinic.

**Methods:**

We will first recruit and conduct qualitative interviews with Veterans with unhealthy alcohol use (n = 20–25) and PC stakeholders (N = 10–15) to understand barriers and facilitators to high-quality alcohol care and use results to refine and hone the multifaceted practice facilitation intervention. Qualitative interviews, analysis, and refinement of the intervention will be guided by the Consolidated Framework for Implementation Research (CFIR). Focus groups with a small sample of PC providers and staff (n = 5–7) will be used to further refine the practice facilitation intervention and assess its acceptability and feasibility. The refined practice facilitation intervention will then be offered in the PC clinic to assess implementation (e.g., reach) and effectiveness (reduced drinking) outcomes based on the RE-AIM framework.

**Discussion:**

This research directly addresses one of the largest public health crises of our time, as alcohol kills more people than opioids and is associated with increased risk of suicide. If successful, this pilot may generate an intervention with far-reaching effects on adverse outcomes experienced by Veterans with unhealthy alcohol use, including increased access to care and suicide prevention.

*Trial registration* Clinicaltrials.gov identifier: NCT04565899; Date of registration: 9/25/2020

**Supplementary Information:**

The online version contains supplementary material available at 10.1186/s13722-022-00300-x.

## Background

Unhealthy alcohol use is common, costly, and leads to adverse negative outcomes including suicide [1, 2]. About one in six Veterans seen in primary care (PC) use alcohol at unhealthy levels [3], which is defined as drinking above recommended limits [4] (more than 7/14 drinks per week for women/men), having any heavy drinking episodes (previously referred to as “binge” drinking; ≥ 4/5 drinks in a day for women/men), or meeting diagnostic criteria for alcohol use disorder (AUD). Unhealthy alcohol use has been linked to over 60 diseases and injuries often seen in PC (e.g., diabetes) and negatively interacts with commonly used medications, further complicating the treatment of these individuals [5, 6]. Evidence-based treatments are available and recommended for delivery in PC settings. They include: brief counseling interventions (BI) for those with unhealthy alcohol use and pharmacotherapy and/or specialized behavioral interventions (e.g., cognitive behavioral therapy) for those with more severe unhealthy alcohol use (i.e., AUD). Implementation in usual care of both types is suboptimal. This protocol describes a mixed-methods pilot study aimed at understanding the barriers and facilitators to evidence-based alcohol-related care in PC and pilot-testing a multifaceted practice facilitation implementation intervention—e.g., providing coaching, identifying clinical champions, and education to a local implementation team.

### Evidence-based alcohol-related care

BIs are traditionally delivered by a PC medical professional (e.g., physician, psychologist). Though content of BIs have varied, consistent elements include individualized feedback on how drinking may adversely impact health and advice to either abstain from or reduce drinking to within recommended limits. For patients with AUD, repeated BI has some benefit, but BI alone is generally insufficient [7]. Thus, for these patients, evidence-based treatment includes pharmacotherapy and/or specialized behavioral interventions (e.g., cognitive behavioral therapy) [8]. FDA-approved medications for AUD—naltrexone, acamprosate, and disulfiram—are promising and effective, yet currently underused in PC [9, 10]. And, though intensive behavioral treatment may be difficult to offer in PC, providers in the Veterans Health Administration’s (VA) integrated behavioral health team (Primary Care-Mental Health Integration; PC-MHI) have expertise in these techniques which could be utilized, and PC providers can facilitate referrals to specialty addictions treatment settings [8, 11].

Increasing access to evidence-based care for unhealthy alcohol use has been a top priority in the VA—the largest integrated healthcare system in the U.S.—since the 1999 Large Health Study of Veterans found that 83% of patients with severe unhealthy alcohol use reported not receiving adequate alcohol care [12, 13]. Since then, VA has used national performance measures to integrate annual screening for unhealthy alcohol use with the Alcohol Use Disorders Identification Test Consumption (AUDIT-C) questionnaire and BI if patients screen positive (AUDIT-C ≥ 5) [14]. Both performance measures are supported by national electronic clinical reminders to support provision and documentation of this care [15]. Evaluations of these efforts have identified high rates of screening (~ 90%) and moderately high rates of documented BI (around ~ 74%), [15, 16] surpassing those of other systems [17–19]. However, higher BI rates are needed, and the treatment landscape is more complicated for those with AUD. While the VA has a clinical guideline recommending further assessment coupled with provision of pharmacotherapy and/or referral to specialty addiction treatment [8, 20, 21] using shared decision making [22] for patients with AUDIT-C scores ≥ 8 (those at greatest risk of AUD [23, 24]) and/or those with documented AUD, most patients with AUD do not receive this care: ~ 12% receive pharmacotherapy, only ~ 26% receive specialty addictions treatment, and receipt of BI has been shown to be associated with decreased likelihood of receiving specialty care (relative to patients with AUD not receiving BI) [25, 26].

Additionally, there have been recent efforts to improve this care in VA, with work focusing specifically on increased pharmacotherapy for AUD via academic detailing with audit and feedback and other implementation efforts (e.g., clinical champions, clinical dashboards) as well as testing an alcohol care management program in primary care [27–29]. Some of this work shows promise by, for example, reducing heavy drinking days in those with AUD [29] and increasing pharmacotherapy rates for those with AUD [27]. However, despite these varied and widespread efforts, both rates of pharmacotherapy and referral to specialty substance use disorder (SUD) care for AUD are still low and disparities exist for the spectrum of alcohol-related care, thus substantial work remains [16, 25, 28, 30, 31]. For example, mixed methods evaluations suggest quality problems with alcohol screening (e.g., non-verbatim verbal screening and miscategorization of unhealthy alcohol use) and multiple barriers to provision of alcohol-related care in PC, [32] including lack of knowledge, experience, and skills; beliefs that provision of alcohol-related care is beyond the scope of PC; concerns about lack of time; and stigma [32]. To improve care, implementation efforts will need to target these barriers, including facilitating the learning/improving of skills (e.g., improve quality of screening), combating stigma, and addressing the anxiety providers convey about time and logistics (i.e., supporting them in effectively integrating this care into their workflow).

### Practice facilitation

Practice facilitation offers a promising avenue for addressing the above noted gaps in high-quality alcohol care. Practice facilitation is an evidence-based multifaceted implementation strategy which has been used in PC to improve the implementation of evidence-based practices across several domains [33, 34] including alcohol-related care [28, 35–37]. Practice facilitation includes multiple components that are used in combination to support clinics in integrating care that addresses gaps aligned with clinic concerns. Components vary but generally include: (1) a practice ‘coach’ who works with clinics on an ongoing basis to help them design workflows and tailor the clinical interventions (e.g., BI) to optimally increase potential for adoption consistent with the goals of the clinic; (2) recruitment of a local implementation team to take ownership of the changes in care; and (3) educational materials and trainings. A recent meta-analysis evaluating practice facilitation’s impact within PC found that PC practices were 2.76 (95% CI 2.18–3.43) times more likely to adopt evidence-based care through practice facilitation [33]. In addition, researchers outside the VA have recently used practice facilitation to integrate alcohol care across 25 PC clinics with initial promising results [38]. Thus, based on its prior success in PC settings (including in VA PC settings) [39] and importantly, practice facilitation’s potential to address the above-mentioned barriers to high-quality alcohol care, this strategy shows promise for improving alcohol-related care in VA.

## Methods

### Aims and overall design

This protocol describes a pilot project designed to refine and evaluate a multifaceted practice facilitation intervention aimed at improving access to and quality of evidence-based alcohol-related care in one VA primary care clinic. Because practice facilitation generally utilizes a variety of implementation strategies, we will conduct initial qualitative interviews to inform intervention design guided by the Consolidated Framework for Implementation Research (CFIR), [40] an implementation framework developed to determine the barriers and facilitators to introducing new practices across multiple clinical contexts.

The PC clinic is in a large, urban VA Medical Center (VAMC), with approximately 100 PC staff and providers, including physicians, residents, fellows, nurses, licensed practical nurses, clinical pharmacists, social workers, and psychologists. The study will be comprised of three aims, conducted over 3 years. The first aim, guided by CFIR [41], will use qualitative methods with key stakeholders to help inform and refine the practice facilitation intervention. We will conduct semi-structured interviews with Veterans (N = 20–25) and PC staff/providers (N = 10–15) to further understand barriers and facilitators to high-quality alcohol care and use results to help develop and hone the practice facilitation intervention. Aim two will include a presentation of the refined practice facilitation intervention to a focus group of VA PC staff and providers (N = 5–7) who will evaluate its acceptability and feasibility. Based on Aim two feedback, the practice facilitation intervention will be revised further. Finally, Aim three will be a pilot test of the refined practice facilitation to evaluate its effectiveness on outcomes guided by RE-AIM (reach, effectiveness, adoption, implementation, maintenance), an implementation evaluation framework [42].

### Procedures, participants, measures, and analyses by study aim

**Aim 1**: *Conduct semi-structured qualitative interviews to identify barriers and facilitators of alcohol-related care and to help refine the practice facilitation intervention*

#### Recruitment and participants

*Clinical stakeholder sample* Clinical stakeholders will include PC providers, staff, and administrators (which comprise approximately 100 providers/staff) affiliated with the Academic Patient Aligned Care Teams (PACT) recruited from a single large VA PC clinic (henceforth called ‘PC clinic’). We will use purposive, snowball sampling methods to recruit clinical stakeholders [43], who will be recruited until data collection reaches saturation of themes [44], consistent with processes routinely used in qualitative data collection where data are collected and analyzed until no new themes emerge (“saturation”) [45–47]. Each participant will also be queried for suggestions of other potential key stakeholders in PC who may offer a different or worthwhile perspective regarding alcohol-related care in PC. We estimate our targeted sample to include 10–15 stakeholders. Interviews will be conducted with individuals in various clinical domains, including PC support staff who administer the AUDIT-C (e.g., licensed practical nurse), and others involved in preventing and intervening on unhealthy alcohol use, including physicians (e.g., attendings, residents), nurses, clinical pharmacists, social workers, and psychologists. We will recruit clinical stakeholders via both email with individual potential participants sent by the PI and presentations by the PI at staff meetings arranged by primary care leadership (e.g., PC section meetings, nurse staff meetings, resident preceptor meetings). We will also followup by telephone and the VA’s instant messaging system [48]. Once potential participants are identified, we will ask if they are interested in participating and, if so, schedule the interview to be conducted by phone. We will subsequently email a standardized information statement briefly explaining the aim of the research prior to consent. Interviews will be conducted by the PI or a research staff member. At the beginning of each interview, clinical stakeholders will be asked to provide verbal informed consent to participate and interviews will follow.

*Veteran stakeholder sample* We will recruit 20–25 Veteran PC patients to understand their experiences in the PC clinic as well as their experiences and preferences related to alcohol care. Similar to clinical stakeholders, Veteran interviews will be conducted until saturation of themes is achieved, which is estimated at 20–25 Veteran stakeholders [44]. We will recruit potentially-eligible Veteran patients through pulling relevant data from VA’s electronic health record (EHR) system (i.e., the Corporate Data Warehouse [CDW]). Recruitment will be targeted, and include Veterans at least 18 years or older who have a documented visit in the preceding 12 months at the participating PC clinic and either documentation of an AUDIT-C score indicating unhealthy alcohol use (scores ≥ 5) and/or a past-year diagnosis of AUD. Exclusion criteria will include the following: documented current (past 6 month) hospice enrollment, documented cognitive impairment (e.g., neurocognitive disorder) over the past 3 years, and/or lack of telephone access. Similar to PC stakeholders, purposive sampling will be used to ensure that results reflect the perspectives of Veterans of varying age, sex, race/ethnicity, and treatment experiences. Based on review of EHR data, we will mail outreach letters weekly to potentially eligible participants (approximately 50 per week). In order to ensure a demographically diverse sample, we will identify blocks of potentially eligible patients balanced on sex and age and approximately one-third each of: underrepresented racial/ethnic groups, AUD diagnosis, or AUDIT-C scores indicating unhealthy alcohol use. We will also examine recent history (past 3 years) of VA interventions for unhealthy alcohol use (e.g., prescribed medications for AUD, attended therapy in specialty substance use clinic) and will purposively sample patients to achieve variability regarding history of prior treatment, to gain perspectives of both types of patient. In the outreach letters, Veterans will be given a phone number to opt out; those who do not opt out in 2 weeks will be called to assess their interest in participating. Veterans deemed not interested will be marked as such and kept on file so they are not re-contacted. Veterans who express interest in the study will be screened by study staff for cognitive impairment using the Blessed Orientation-Memory Concentration Exam (BOMC [49];), a brief screening interview that can identify individuals who may have cognitive impairment that has gone un-diagnosed in the EHR. If the Veteran is interested and is above threshold on the BOMC interview (score between 0 and 10), they will be deemed eligible, mailed a study information sheet, and invited to schedule a phone interview. Trained research staff will conduct the qualitative interview. Prior to the interview, study staff will review the information sheet and obtain verbal informed consent. Participants will be compensated $35 for their time. All proposed procedures have been approved by the local VA IRB.

#### Data collection

Qualitative interviews will be conducted via phone. All interviews will include open-ended and suggested semi-structured follow-up questions, developed using the 5 broad domains of the CFIR (Outer Setting, Inner Setting, Intervention Characteristics, Individual Characteristics, and the Process of Implementation) [40] and by using guidance from the open access CFIR website and prior work by the authors. The PC stakeholder interview includes questions regarding their role in the PC clinic, experiences and thoughts on treating patients with unhealthy alcohol use, and feedback on ideas about how we could improve/support provision of care for unhealthy alcohol use. The Veteran interview guide includes questions regarding their general experience in the PC clinic, their alcohol treatment-seeking history, conversations they have had with PC providers about alcohol, role of the VA/health system in treating unhealthy alcohol use, and interest in various evidence-based alcohol treatment options.

#### Data analysis

All interviews will be audio recorded, transcribed verbatim, and verified by two members of the study staff who are trained transcriptionists. We will use a de-naturalized transcription process, which represents an attempt at verbatim accuracy—with redactions to anonymize identifying information such as names—while favoring the context of the exchange over the structure. Data will be analyzed using the Rapid Assessment Process (RAP) [50], which is an “intensive, team-based qualitative inquiry using triangulation, iterative data analysis and additional data collection to quickly develop a preliminary understanding of a situation from the insider’s perspective.” RAP is often used in health services research when the goal is to use qualitative data to make real-time modifications to an implementation strategy [51]. Thus, qualitative analysis will be conducted iteratively with the CFIR model. First, as discussed above, all stakeholder interview guides will be informed by CFIR domains. We will then analyze data using RAP, which will extract data by question and place it by domain into a summary template by study staff. The PI, research assistant, transcriptionists, and a qualitative methods expert will meet weekly to review each summary template and come to a consensus should there be coding discrepancies. Data from each summary template will then be transferred to a data matrix. At the end of data collection, all results in the matrix will be reviewed by the PI and the investigator team to understand themes (barriers and facilitators of care) and how they relate to the CFIR domains and subdomains. Themes (barriers/facilitators) will then be summarized within their CFIR domain/subdomain and subsequently will help to guide the next phase of the study (refining the practice facilitation).

#### Refining the practice facilitation intervention

After analyzing and summarizing the top barriers and facilitators of care from qualitative interviews (e.g., the most frequently mentioned barriers) the investigator team will meet to help finalize components of the practice facilitation for presentation in Aim 2. Initial components of the practice facilitation intervention are based on a recent body of implementation work, including work by co-authors, aiming to integrate and improve alcohol-related care in PC [35, 52] and specialty medical settings (e.g., Liver clinics) [38, 53, 54] and include: a practice coach to support changes in clinic workflow (CFIR inner setting) and provide content training in evidence-based alcohol-related care and stigma reduction (CFIR individual characteristics and inner setting), and recruitment of a local implementation team to support dissemination of knowledge and care processes (CFIR implementation process). The practice coach will also support optimization of existing EHR tools to support provision and documentation of alcohol-related care (CFIR inner setting and implementation process). Additionally, while refining the practice facilitation, we will consult the CFIR-ERIC (Expert Recommendations for Implementing Change) match tool [55], which was developed via expert panel to help suggest various implementation strategies matched to each CFIR subdomain. For example, if our qualitative findings suggest that stigma and lack of knowledge regarding effective BI delivery are significant barriers to care, our practice facilitation intervention will include the practice coach offering tools to overcome stigma (e.g., educational handout reframing how the spectrum of unhealthy alcohol use affects health) and improve knowledge about evidence-based BIs for reducing unhealthy drinking. Proposed timeline and components of the practice facilitation are described below in further detail (Aims 2 and 3).

**Aim 2:** *Assess feasibility and acceptability of the practice facilitation intervention*

#### Recruitment and participants

We will recruit a focus group of PC staff and providers (N = 5–7) to assess the acceptability and feasibility of the refined practice facilitation strategy, including evaluation of any educational materials we plan on providing during the pilot study proposed for Aim 3. The project’s facilitator will run the focus groups and serve as the content expert on effective evidence-based alcohol-related care. The facilitator will undergo facilitation training via multiple methods, including by participating in formal facilitation training provided by VA (“Behavioral Health QUERI Implementation Facilitation Training”), guided readings (e.g., *Implementing Evidence-Based Practice in Healthcare: A Facilitation Guide* [56;]) and videos regarding facilitation and evidence-based alcohol-related care, [57, 58] and meetings with the PI and co-investigators. The facilitator will meet 2–3 times with the focus group (see Table 1) as a way to practice delivering the material and to assess whether the ideas and strategies presented are acceptable and feasible in the PC clinic.

**Table 1:** Practice facilitation components

Component	Description		
Facilitator	The facilitator will facilitate the PC clinic’s development of the implementation plan and serve as a content expert who will train staff/providers on evidence-based care for unhealthy alcohol use		
Clinic Champion (Aims 2 & 3)	The facilitator will meet and work with a Clinic Champion(s) to recruit and present the study to PC staff/providers		
Aim 2: Focus Groups with small sample of PC clinical stakeholders (N = 5–7) (Acceptability/Feasibility assessment)	Focus Group Meetings: PC staff and providers interested in helping evaluate acceptability and feasibility (Aim 2) of the practice facilitation will participate in 2–3 successive focus groups, which will be scheduled flexibly around their availability and workflow. Meetings will cover: desired changes in clinic workflow, review ideas for content training in evidence-based alcohol-related care and stigma reduction, recruitment of a local implementation team to support dissemination of knowledge and care processes, and support optimization of existing EHR tools to aid in provision and documentation of alcohol-related care. Meetings will take place over a 4-month timespan. Proposed meeting goals/topics are summarized below		
	*Proposed topics to be covered during Aim 2 focus groups*		
	• Introduce facilitation and didactics (basic concepts regarding best practices for unhealthy alcohol use) Provide a facilitation example and conduct a current state assessment (what is happening regarding care for alcohol use now?) Set goals (what do we wish were happening?)	• Create an initial workflow plan Discuss whether piloting a workflow plan is feasible Review concept of assessing successes and challenges in piloting plan and making adjustments Present sample educational materials and garner feedback	• Identify a local implementation team Identify process for audit and feedback using EHR Assess acceptability and feasibility of the practice facilitation materials via notes, observations, provider self-report, and debriefing interview
	Patient-centered Materials: If education is deemed a top barrier, the facilitator will share patient-centered materials, including a handout on “old” and “new” approaches to thinking about alcohol and health as well as a YouTube video, developed by co-author Dr. Williams, presenting this reframe. Materials will be available for PC staff and providers to use with patients and will be designed to help train staff. Materials for assessment of and referral to treatment for specialty SUD care will also be included		
	Informatics Tools in EHR: Across all meetings, the facilitator will review three main informatics tools PC providers can use when preventing and intervening on unhealthy alcohol use: 1) Electronic Clinical Reminder. Description of BI when patient screens positive (AUDIT-C) 2) Order Set. This tool walks the clinician through several different ways of approaching treatment for unhealthy alcohol use including provision of a prescription for AUD pharmacotherapy 3) Consult Menu. Easily enables a referral to specialty SUD treatment at the VA medical center		
Aim 3: Pilot test of the Practice Facilitation Intervention with Full PC Clinic (Active Implementation)	The facilitator will administer the final version of the practice facilitation intervention via 2 initial welcome group meetings followed by weekly or biweekly meetings with PC providers/staff identified as the local implementation team (for approx. 5 months). Meetings will troubleshoot ongoing issues for providers/staff and include performance monitoring and feedback		
	Performance Monitoring and Feedback via CDW: Real-time data will be fed back to PC providers regarding rates of patients with unhealthy alcohol use identified, rates of brief intervention offered, rates of consults to PC-MHI and SUD treatment, and rates of prescribed AUD pharmacotherapy		

The site for recruitment is the same site from Aim 1. We will recruit PC staff and providers with the help of PC leadership who will act as study liaisons and help us identify a “Clinic Champion(s)”, a provider(s) who will advocate for participation in the study. The Clinic Champion(s) and liaisons, as well as feedback we received from our Aim 1 qualitative interviews, will help identify staff and providers eligible and willing to participate. Eligible participants include PC staff who conduct alcohol screening and providers who practice at the PC clinic at least 1 day per week. No other exclusion criteria will be applied; thus, providers who participated in Aim 1 interviews will be eligible for participation in Aim 2. We will follow-up with individuals interested in participating via email to introduce the PI and the study and ask if they could speak briefly with study staff about participating in focus groups related to addressing unhealthy alcohol use. Focus groups will either be virtual (e.g., via Microsoft Teams) or in-person, depending on guidance related to the COVID-19 pandemic. At the end of the focus groups, we will assess acceptability and feasibility of the practice facilitation (projected to run over a span of 4 months; see Table 2).

**Table 2:** Phases of the practice facilitation intervention

Phase	Acceptability/Feasibility assessment	Active implementation	Post-implementation
Study aim	Aim 2	Aim 3	Aim 3
Time	4 months	5 months	3-, 6-, 12-months post Active Implementation
Description	Facilitator identifies & meets with Clinic Champion(s) who helps recruit & present study to PC staff/providers (2 months) Present facilitation ideas and materials to small group of PC staff/providers; assess acceptability & feasibility of the refined practice facilitation (1–2 months) Modify intervention in preparation for Aim 3 full clinic pilot	Deliver the finalized practice facilitation Weekly or biweekly meetings between the facilitator and PC staff/providers over 5 months	Measure & analyze implementation outcomes at 3-, 6-, and 12-months post active implementation Analyze effectiveness outcomes at 12-months post active implementation

#### Data collection, outcomes, and analysis

*Focus group qualitative data collection* Participants will meet with the facilitator 2–3 times to iteratively refine the practice facilitation intervention components and assess their acceptability and feasibility. Each focus group will last 1–2 h and cover slightly different content, described in detail in Table 1.

*Outcomes: acceptability and feasibility* Acceptability is the extent to which the use of practice facilitation to integrate alcohol-related care is agreeable, appealing, and satisfactory to the clinic, and feasibility is defined as the extent to which practice facilitation can be successfully used or carried out at the PC clinic where the study is being conducted. Acceptability and feasibility information will be collected qualitatively and quantitatively. First, notes and observations will be summarized from each focus group meeting (see below) by a research assistant and/or PI. Notes will then be coded to indicate level of acceptability and feasibility of the practice facilitation. Second, PC participants will fill out two brief self-report measures at the end of Aim 2, indicating level of acceptability (4 items) and feasibility (4 items) of the practice facilitation (see Additional file 1). Items are derived from the Acceptability/Feasibility of Intervention Measure, developed for implementation science research and shown to have good content/discriminant validity and reliability in a large sample of providers [59]. Last, study staff will conduct an optional one-on-one debriefing interview at the end of Aim 2 to gauge PC staff/providers views on participating in the practice facilitation that will tap into acceptability and feasibility constructs (see Additional file 1).

*Qualitative and quantitative analysis of focus group design meeting data* We will use rapid data analytic techniques described above [60, 61] to quickly and efficiently code for acceptability and feasibility of the material and then iteratively revise the practice facilitation intervention. Each focus group will be audio recorded, and a research assistant and/or PI will also take notes in real time to help record feedback and discussion. Any notes written by participants and the facilitator will be collected. Audio files will be transcribed by a study transcriptionist and we will use the transcription and our notes during study staff meetings to summarize feedback/recommendations given, highlighting feedback related to acceptability and feasibility, and resolve any differences that may occur. This process will take place after each focus group meeting. Numeric data from the Acceptability/Feasibility of Intervention Measure will be averaged by scale, with higher scores indicating greater acceptability and feasibility. Cut-off scores for interpretation are not yet available, but we will define good acceptability and feasibility as a minimum rating of 4 ("agree”) on a 0–5 (“Completely Disagree”–“Completely Agree”) Likert scale for all 8 items. Last, debriefing interviews at the end of the last focus group will be summarized in the same manner as described above (e.g., audio-recorded, transcribed, coded for acceptability/feasibility). The summarized qualitative feedback and the numeric quantitative feedback will be presented to co-investigators for their feedback and suggestions, and subsequently, the findings will be used to revise the practice facilitation if needed.

**Aim 3:** *Pilot test of the practice facilitation to understand whether this implementation strategy has the potential to improve care*

#### Recruitment and participants

Aim 3 will be conducted at the clinic level, and PC providers and staff affiliated with the Academic PACTs (i.e., the PC clinic) will be recruited to join a local implementation team in the same manner as recruitment occurred for Aim 2 (e.g., email from the PI, presentations in PC staff meetings, direct messages from the PI to staff, discussions with the Clinic Champion(s)); providers/staff who took part in Aims 1 and/or 2 will be encouraged and eligible to take part in Aim 3. Providers and staff interested in joining the local implementation team will meet regularly with the facilitator, take ownership of the changes in alcohol-related care, and support dissemination of alcohol-related knowledge and care processes to the clinic. Table 2 and the bottom of Table 1 provides the projected content and outline of the practice facilitation intervention, which is expected to occur over a span of 5 months. The facilitator will first conduct two initial ~ one hour meetings with the local implementation team (either virtually or in-person, depending on the COVID-19 pandemic), to introduce the finalized version of the practice facilitation. The facilitator will then conduct weekly or biweekly meetings with the implementation team and communicate regularly via email with the Clinical Champion(s) for ongoing support. Ongoing weekly or biweekly meetings will most likely include the delivery of performance monitoring and feedback, based on “Plan Do Study Act” or “PDSA” cycles, to help providers identify gaps in alcohol care and test solutions to optimize care quality [52]. With guidance from previous implementation work [52], performance monitoring and feedback will include: weekly proportion of PC patients screened for unhealthy alcohol use, rates of PC patients who screened positive for unhealthy alcohol use (AUDIT-C ≥ 5), rates of BI offered for those screening positive, rates of referrals to PC-MHI for those screening positive, rates of consults to the specialty SUD clinic for both those screening positive and those at higher risk of developing an AUD (AUDIT-C ≥ 8), rates of prescribed AUD pharmacotherapy for those with an AUDIT-C ≥ 8, and monthly specialty SUD treatment initiation (patient attended ≥ 1 appointment). This information will be presented and discussed with PC providers at the above-mentioned weekly or biweekly meetings throughout the Active Implementation phase. We will use administrative data from the EHR to populate performance monitoring and feedback forms. Feedback data will be calculated at the provider and clinic-level and framed positively to either encourage providers to continue providing this care or to adopt this care more frequently.

#### Pilot study outcomes and data sources

*Evaluation of implementation and clinical outcomes* Aim 3 implementation-specific outcomes were derived and guided by the RE-AIM (Reach, Effectiveness, Adoption, Implementation, and Maintenance) evaluative framework, which is often used to guide implementation evaluations [42]. Specifically, the Aim 3 pilot will assess reach, effectiveness, adoption, and maintenance domains. All outcomes are defined and elaborated on below.

*Reach* Reach of alcohol-related care will be assessed at the patient level using EHR data, pulled from VA’s CDW. Reach outcomes will be expressed as a rate, with the denominator defined as all eligible Veterans with a visit to the PC clinic during Active Implementation (e.g., the denominator to understand rates of alcohol screening will include all Veterans with a PC clinic visit, the denominator to understand rates of BI will include those with an AUDIT-C ≥ 5). The numerator for each reach outcome is as follows: the number of Veterans with unhealthy alcohol use who: had a documented BI, engaged in specialty SUD treatment (inpatient and outpatient clinic visits for SUD treatment encounters with an accompanying AUD diagnosis), engaged in PC-MHI, or received pharmacotherapy for AUD (any filled prescription for FDA-approved medications: acamprosate, disulfiram, or oral/injectable naltrexone). Rates will be assessed in the 30 days following a positive screen. Denominators will also be derived to represent specific subpopulations (e.g., women). Rates at the start of Active Implementation and at 3- and 6-months post Active Implementation will be calculated to examine change in Reach outcomes over time (see Table 2 for timeline of implementation).

*Adoption* Adoption is identified as the percentage of providers from the PC clinic who deliver alcohol-related care when a Veteran screens positive for unhealthy alcohol use. Adoption will be measured using both EHR data collected during performance monitoring/feedback and by provider self-report. Specifically, we will use data available in the EHR to examine delivery of BIs, referrals placed to PC-MHI or specialty SUD treatment, and pharmacotherapy provided for AUDs at the provider and clinic level. Rates of alcohol-related care at the start of Active Implementation and at 3- and 6-months post Active Implementation will be used to measure whether adoption rates of alcohol-related care increased over time. In addition, we will collect brief self-report questions from PC providers to determine whether providers have adopted the suggested evidence-based components of alcohol-related care (e.g., shared decision making, patient-centered discussions, setting goals with patients) [8, 62]. Self-report items were derived and adapted from the Shared Decision Making Questionnaire [62] and VA/DoD SUD Clinical Guidelines [8]. Items are rated on 5-point Likert scales, with higher scores reflecting better shared decision making and implementing treatment in line with SUD clinical guidelines. Verbal consent will be obtained and those that consent will be asked to fill out the self-report items at the start of the Active Implementation phase, at the end of the Active Implementation phase, and 3- and 6-months post practice facilitation (Post-Implementation phase).

*Maintenance* Maintenance is defined as the extent to which evidence-based alcohol-related care becomes routine and part of the PC culture (care sustained over time). Reach and adoption outcomes using EHR data will be assessed at 12-months post Active Implementation to examine whether high-quality alcohol care was sustained.

*Effectiveness* Effectiveness is defined as the change in prevalence of unhealthy alcohol use at 12-months post-intervention as measured by AUDIT-C scores. This variable will also be expressed as a rate with the denominator defined as all Veterans seen in the PC clinic during Active Implementation. The numerator will be the number of Veterans with an encounter in the PC clinic who screened positive for unhealthy alcohol use (AUDIT-C ≥ 5) during Active Implementation and at 12-months post facilitation intervention. See Data analysis for explication on how we plan to assess change in AUDIT-C over time.

#### Data analysis

*Implementation outcomes* Reach outcomes will be reported descriptively (e.g., percentage of patients with an AUDIT-C ≥ 5 who were offered a BI or pharmacotherapy for AUD) and compared pre- and post-implementation (at 3- and 6-months) to assess whether reach of alcohol care increased over time. Adoption outcomes via EHR and self-report will also be descriptive, and include percentages, frequencies, and means (SD) of alcohol-related care by provider and clinic to understand whether adoption of high-quality alcohol-related care increased over time. Descriptives will be examined and compared at the start of Active Implementation and at 3- and 6-months post Active Implementation. Reach and adoption rates will again be examined at 12-months post Active Implementation and compared to rates at 3- and 6-months to see if high-quality alcohol-related care was sustained (i.e., maintenance).

*Effectiveness* Effectiveness will be assessed via change score analysis, where the Veterans visiting the intervention site will serve as their own control, and we will investigate whether Veterans with an encounter in the PC clinic during Active Implementation reduced their drinking 12-months post Active Implementation using AUDIT-C scores. Change in the outcome (AUDIT-C scores) will be modeled using a regression model with difference in the outcome (AUDIT-C scores at Time 2 – AUDIT-C scores at Time 1) as the predicted variable. The significance of the intercept of this model will provide a test of average change. We will then include Time 1 scores as a predictor to adjust for initial severity of the outcome in the estimation of average change. In this model, a negative intercept would be indicative of change in drinking such that AUDIT-C scores at 12-months post Active Implementation are on average lower. Additionally, we will also be able to assess whether there was meaningful change in AUDIT-C scores over time by calculating a Reliable Change Index (RCI) [63]. The RCI can aid in understanding whether a decrease in AUDIT-C scores over time is clinically meaningful.

## Discussion

The present protocol describes a pilot study aiming to improve PC-based alcohol-related care using practice facilitation, an evidence-based implementation strategy. This research directly addresses one of the largest public health crises of our time, as alcohol kills approximately twice as many people as opioids [64] and is associated with increased risk of suicide [2]. To our knowledge, this is the first study to use practice facilitation and larger system-level implementation strategies (e.g., performance measures) to try and *improve* the spectrum of alcohol-related care in a primary care setting. A recent large, multi-site implementation trial (the Sustained Patient-centered Alcohol-Related Care (SPARC) trial) tested whether practice facilitation increased rates of alcohol-related care in primary care settings [35, 65, 66]; however, the SPARC trial focused on implementing alcohol-related care from the ground up (not improving already existing care) as well as implementing integrated behavioral health care (e.g., screening for depression, drug use). Thus, the present study will extend this work by hopefully addressing how integrated alcohol-related care can be improved upon and how these improvements can be sustained. In addition, the present study builds on the substantial investment VA has made to ensuring that Veterans with unhealthy alcohol use receive the care they need and may have far-reaching effects on adverse outcomes (e.g., suicide prevention) should the implementation strategy be effective.

### Strengths and limitations

Despite the potential impact of this pilot study, there are both strengths and limitations worth mentioning. A major strength of this project is the collection and assessment of perspectives from key stakeholders (clinical and Veteran) on comprehensive alcohol care (i.e., screening, BI, prescribing AUD medications, referral/warm-hand-off to specialty treatment) to inform and hone a practice facilitation intervention aimed at improving care while meeting their goals and needs. Moreover, this project extends previous qualitative work with Veterans [67, 68] by assessing demographically diverse Veterans on their perceptions of barriers and facilitators of comprehensive alcohol-related care in PC settings, which both builds on prior work while offering new contribution. In addition, we believe Aim 2 focus groups are a strength, as they offer efficiency of gathering many opinions, stimulate discussion that may not naturally occur in one-on-one interviews, and may also provide opportunities to identify views that fall outside “the norm” or outliers, which can skew findings if not identified [69]. Last, the Aim 3 pilot study will be a tailored facilitation intervention in the PC clinic aiming to improve care that is needed and valued highly by VA. However, despite these strengths, there are several limitations of the present work. First, it is possible that limiting the Aim 1 qualitative study to stakeholders at one VA medical center could limit the richness of our findings, as PC clinics at other VAs may have distinct barriers/facilitators to alcohol-related care, which would necessitate different modifications to an implementation intervention. Additionally, scheduling focus groups and facilitation meetings at a time easily attended by many may be difficult and PC participants may be influenced by other’s opinions or not feel confident in voicing their views. Given these limitations, we will make every effort to conduct focus groups at a time (e.g., before/after work hours) and place (e.g., virtual and/or in-person) amenable to all/most members and we will inform participants that they can reach out to us after meetings (e.g., via email) if they want to express further opinions about the practice facilitation intervention. Last, the major limitation of our Aim 3 pilot study is that it will only include one PC clinic, thus implementation outcomes may not easily generalize to other PC clinics and provider self-report assessments may be biased, as self-report assessments will only be collected by PC providers who consent to us collecting this data. Despite this, we believe the strengths outweigh the limitations as this will be the first study to use a tailored evidence-based implementation strategy to improve the spectrum of alcohol care in a VA PC clinic. As discussed briefly above, we also believe that this study will complement what has been accomplished thus far in VA and provide a roadmap for testing these aims on a larger scale.

### Conclusion

Alcohol use is a leading risk factor for disability and death in Veterans [1, 70]. Evidence-based care for unhealthy alcohol use is recommended in PC, [8, 9, 20, 21, 71–73] however, most patients with unhealthy alcohol use do not receive these treatments [74]. The VA was a pioneer in implementing alcohol-related care in PC, [13, 18, 75] but implementation gaps point to a need for innovative methods to improve access and quality [76, 77]. Practice facilitation is an evidence-based multilevel implementation strategy [33] that holds promise for closing quality gaps in VA’s alcohol-related care. The present study will be the first to test a tailored practice facilitation intervention to improve the spectrum of PC-based alcohol care. Implementation and clinical results from our pilot study will have the potential to inform future work aiming to improve the quality of and access to alcohol-related care in PC clinics across the VA healthcare system.

## Supplementary Information

**Additional file 1.** Acceptability and feasibility of intervention measure and the primary care provider debriefing interview.

## Data Availability

Not applicable. This paper does not include any data as it is a protocol paper. When data is collected, we ask that readers please request it from the lead author.

## Abbreviations

AUD: Alcohol use disorders
AUDIT-C: Alcohol Use Disorders Identification Test-Consumption
BI: Brief alcohol intervention
CDW: Corporate Data Warehouse
CFIR: Consolidated Framework for Implementation Research
DoD: Department of Defense
EHR: Electronic health record
ERIC: Expert Recommendations for Implementing Change
FDA: U.S. Food and Drug Administration
NIAAA: National Institute on Alcohol Abuse and Alcoholism
PI: Primary Investigator
PC: Primary care
PC-MHI: Primary Care-Mental Health Integration
RE-AIM: Reach, effectiveness, adoption, implementation, maintenance
SUD: Substance use disorders
VA: Veterans Health Administration
