# Supplementary material for: Using practice facilitation to improve alcohol-related care in primary care: a mixed-methods pilot study protocol
Source: Addict Sci Clin Pract. 2022 Mar 14;17:19. doi: 10.1186/s13722-022-00300-x (PMC8919159; doi:10.1186/s13722-022-00300-x)
Supplement: Supplementary file 1 — Additional file 1. Acceptability and feasibility of intervention measure and the primary care provider debriefing interview. [file 13722_2022_300_MOESM1_ESM.docx]

Acceptability and Feasibility of Intervention Measure

GENERAL INSTRUCTIONS: Fill out the below items related to the group meetings you had with the practice facilitator about alcohol-related care in primary care. As a reminder, completing these items are voluntary and your answers are anonymous and confidential.

**Acceptability of Intervention Measure (AIM)**

|  | Completely disagree | Disagree | Neither agree nor disagree | Agree | Completely agree |
| --- | --- | --- | --- | --- | --- |
| 1. This alcohol-related practice facilitation meets my approval. | ➀ | ➁ | ➂ | ➃ | ➄ |
| 2. This alcohol-related practice facilitation is appealing to me. | ➀ | ➁ | ➂ | ➃ | ➄ |
| 3. I like the alcohol-related practice facilitation. | ➀ | ➁ | ➂ | ➃ | ➄ |
| 4. I welcome the alcohol-related practice facilitation. | ➀ | ➁ | ➂ | ➃ | ➄ |

**Feasibility of Intervention Measure (FIM)**

|  | Completely disagree | Disagree | Neither agree nor disagree | Agree | Completely agree |
| --- | --- | --- | --- | --- | --- |
| 1. The alcohol-related practice facilitation seems implementable. | ➀ | ➁ | ➂ | ➃ | ➄ |
| 2. This alcohol-related practice facilitation seems possible. | ➀ | ➁ | ➂ | ➃ | ➄ |
| 3. This alcohol-related practice facilitation seems doable. | ➀ | ➁ | ➂ | ➃ | ➄ |
| 4. Materials from the alcohol-related practice facilitation seems easy to use. | ➀ | ➁ | ➂ | ➃ | ➄ |

**Primary Care Provider Debriefing Interview**

INSTRUCTIONS

*(Administer after the last focus group of Aim 2).* We would like to understand how you viewed the acceptability and feasibility of the practice facilitation - both the content of the materials and the meetings with the facilitator. As a reminder, this interview is voluntary, anonymous, and confidential.

1. What was your experience like regarding meeting with the facilitator? (We are interested in both positive and negative experiences)
2. How satisfied were you with these meetings?
   - What could be better or more satisfactory?
3. How satisfied were you with the education materials?
   - What could we do to improve them?
4. How suitable did you think the education materials were for a primary care setting?
   - What would make them more suitable or useful to a primary care setting?
5. What else do you think is important for us to know about the practice facilitation before inviting the full primary clinic at VAPHS to participate?

Thank you very much for answering our questions. The information you provided is extremely valuable and useful to us.
